# Heterozygous mutations in factor H aggravate pathological damage in a stable IgA deposition model induced by *Lactobacillus casei* cell wall extract

**DOI:** 10.3389/fimmu.2024.1368322

**Published:** 2024-03-15

**Authors:** Jingyi Li, Yaping Dong, Feifei Chen, Hongyu Yang, Pei Chen, Hongyu Li, Sufang Shi, Xujie Zhou, Li Zhu, Yuemiao Zhang, Lijun Liu, Xinfang Xie, Feng Yu, Jing Jin, Jicheng Lv, Hong Zhang

**Affiliations:** ^1^ Renal Division, Peking University First Hospital, Beijing, China; ^2^ Institute of Nephrology, Peking University, Beijing, China; ^3^ Key Laboratory of Renal Disease, Ministry of Health of China, Beijing, China; ^4^ Key Laboratory of Chronic Kidney Disease Prevention and Treatment (Peking University), Ministry of Education, Beijing, China; ^5^ State Key Laboratory of Vascular Homeostasis and Remodeling, Peking University, Beijing, China; ^6^ Department of Nephrology, The First Affiliated Hospital of Xi’an Jiaotong University, Xi'an, China; ^7^ Department of Nephrology, Peking University International Hospital, Beijing, China; ^8^ Northwestern University Feinberg School of Medicine, Division of Nephrology, Chicago, IL, United States

**Keywords:** IgA nephropathy, complement factor H, mouse model, the alternative complement pathway, proteinuria

## Abstract

**Introduction:**

Activation of complement through the alternative pathway (AP) has a key role in the pathogenesis of IgA nephropathy (IgAN). We previously showed, by intraperitoneal injection of Lactobacillus casei cell wall extract (LCWE), C57BL/6 mice develop mild kidney damage in association with glomerular IgA deposition. To further address complement activity in causing glomerular histological alterations as suggested in the pathogenesis of IgAN, here we used mice with factor H mutation (FH^W/R^) to render AP overactivation in conjunction with LCWE injection to stimulate intestinal production of IgA.

**Methods:**

Dose response to LCWE were examined between two groups of FH^W/R^ mice. Wild type (FH^W/W^) mice stimulated with LCWE were used as model control.

**Results:**

The FH^W/R^ mice primed with high dose LCWE showed elevated IgA and IgA-IgG complex levels in serum. In addition to 100% positive rate of IgA and C3, they display elevated biomarkers of kidney dysfunction, coincided with severe pathological lesions, resembling those of IgAN. As compared to wild type controls stimulated by the same high dose LCWE, these FH^W/R^ mice exhibited stronger complement activation in the kidney and in circulation.

**Discussion:**

The new mouse model shares many disease features with IgAN. The severity of glomerular lesions and the decline of kidney functions are further aggravated through complement overactivation. The model may be a useful tool for preclinical evaluation of treatment response to complement-inhibitors.

## Introduction

IgA nephropathy (IgAN) is the most common form of primary glomerular disease worldwide (1). More than half of the patients will eventually progress to end-stage renal disease (2). The hallmark of IgAN is IgA deposition in the mesangial area, frequently accompanied by the co-deposition of complement C3. The pathophysiology of the disease is summarized in a four-hit model. Central to the pathogenesis is an increase in circulating galactose deficient-IgA1 [Gd-IgA1] (hit 1). These abnormally glycosylated IgA1 molecules form immune complexes with autoantibodies in the systemic circulation (hit 2). These immune complexes eventually deposit in the mesangial area of the glomerulus (hit 3) to trigger inflammatory responses, including complement activation leading to tissue damage (hit 4) (3). However, the existing animal models only reflect the disease process to a limited extent although they had made a great contribution to the research of IgA nephropathy (4–6). We previously reported a mouse model with intraperitoneal injection of *Lactobacillus casei* cell wall extract (LCWE) emulsified with Complete Freund’s Adjuvant (CFA) to cause chronic inflammation. In this model, we noted a continuous and stable deposition of IgA in the glomerular mesangial areas, with high circulating levels of IgA and IgA-IgG complexes (7). While this model showed consistent IgA deposition, complement C3 co-deposition is rare in this model and there is only mild kidney damage.

Large international genome–wide association studies (GWAS) identified variants of complement factor H-related (CFHR) genes being associated with IgAN. The genome–wide significant effect of CFHR3,1 gene deletion to reduce the risk for IgAN qualifies activators and regulators of the alternative pathway (AP) as major players in the pathogenesis of the disease (8). However, the precise role of factor H and CFHRs in IgAN progression remains unclear. As an important regulator of the AP, factor H prevents the excessive activation of complement (9). A point mutation (W1206R) of factor H results in an increased binding to C3b, impairing its interaction with host cells (10). Mice harboring this mutation have a higher propensity of localized complement activation to cause complement-mediated kidney injury. Homozygous mice carrying this mutation develop severe thrombotic microangiopathy (TMA). Although heterozygous animals stay largely normal, they are susceptible to complement activation in the kidney.

In the current study, we exploited the factor H heterozygous mice (FH^W/R^) by challenging them with LCWE. LCWE primes the animals for intestinal IgA production, in causing IgA deposition in the kidney. When combined with FH^W/R^ that predispose the animals for complement overactivation, the model developed diverse histological lesions in the kidney that closely resemble IgAN’s MEST-C alterations.

## Materials and methods

### Mouse procedures

All mice were raised and maintained under specific pathogen-free (SPF) conditions. All experiments were performed in accordance with local guidelines for laboratory animal care and the study were approved and supervised by the Laboratory Animal Care and Use Committee of Peking University First Hospital (No. 202103).

Complement factor H heterozygous mutant mice (FH^W/R^) and wild type mice (FH^W/W^) were generated by mating FH^W/R^ mice with C57BL/6J mice (FH^W/W^) (10). In this study, only male mice were included.

The extraction of *Lactobacillus casei* cell wall extract (LCWE) and its administration were prepared as previously described (7). The FH^W/R^ mice were randomly allocated to three groups: PBS group (FH^W/R^-Buffer), low dose LCWE emulsified with CFA group (0.1ug/g, FH^W/R^-LCWE^Low^), and high dose LCWE emulsified with CFA group (0.4ug/g, FH^W/R^-LCWE^High^). FH^W/W^ mice were randomly assigned to one of the two groups: PBS group (FH^W/W^-Buffer), and high dose LCWE emulsified with CFA group (0.4ug/g, FH^W/W^-LCWE^High^). The FH^W/W^-Buffer is only used as a control for western blot.

Intraperitoneal injection (i.p.) of LCWE commenced when the mice reached 8 weeks of age. Collections of serum and urine samples started at 2 months of age. Blood samples were obtained from the orbital vein, while urine samples were collected using metabolic cages. In order to observe the sequential changes of pathological damage in the model, mice in each group were sacrificed at 18, 26, and 30 weeks of age.

### Histology, immunofluorescence, immunohistochemistry and electron microscopy

Paraffin-embedded kidney specimens 1.5µm in thickness were stained with periodic acid-Schiff (PAS) using standard protocols for morphological analysis. Pathological lesions were evaluated using PAS staining. As there is no established universal standard for renal lesions in IgA nephropathy model mice, we assessed the lesions (MEST-C) according to the Oxford classification of IgA nephropathy (**Supplementary Table S1**). In addition, we evaluated arteriolar lesions (A) including wall thickening and onion-like lesions.

For immunofluorescence staining, 2-µm thick cryostat frozen kidney sections were incubated for 1 hour at 37°C with Alexa Fluor 488-conjugated goat antibodies specific for mouse IgA (SouthernBiotech, 1040-30, 1:200, 5ug/ml), FITC-conjugated goat anti-mouse C3 antibody (MP Biomedicals, 0855500, 1:1000, 4ug/ml), FITC-conjugated goat anti-mouse C1q antibody (HycultBiotech, HM-1096F, 1:500, 2ug/ml), rabbit anti-mouse Bb antibody (Genetex, GTX81508, 1:200, 5ug/ml) and rabbit anti-mouse MBL antibody (abcam, ab190834, 1:100, 10ug/ml). For the staining of the latter three antibodies, after washing three times with PBS, incubate the matching fluorescent secondary antibodies at 37°C for 1 hour. Nuclei were stained with DAPI (Abcam). The images were collected using a fluorescent microscope (*Leica*, Germany). The intensity of staining was evaluated and scored on a scale of - to +++: -, no staining and trace staining; +, mild staining; ++, moderate staining; and +++, high staining on a high-power field. For IgA and C3 colocalization staining, we used the another C3 antibody (abcam, ab200999, 1:100, 10ug/ml).

Immunohistochemistry staining was performed for CFH and C5b-9. First, 2-µm thick paraffin embedded renal tissues were deparaffinized in xylene and rehydrated in grading alcohols, then sections were treated by appropriate antigen retrieval methods for 10 minutes at 37°C, including proteinase k (ZSGB-BIO, ZLI-9016, 0.5mg/ml) and proteinase XXIV (Sigma, P0652, 0.5mg/ml). After quenching endogenous peroxidase activity for 10 minutes at room temperature, sections were incubated with primary antibody for 1 hour at 37°C. All the antibodies’ optimal dilutions were predetermined. For CFH (Merck, 341276, 1:5000), after incubation with fluorescent secondary antibodies, nuclei were stained with DAPI (Abcam). And for C5b-9 (abcam, ab55811, 1:250, 20ug/ml), after incubating with the HRP-conjugated secondary antibody, the sections were developed using a fresh hydrogen peroxide plus 3-3-diaminobenzidine tetrahydrochloride solution for an appropriate duration. Finally, the sections were counterstained with hematoxylin, anti-blue with lithium carbonate, dehydrated and cleared in alcohols and xylene. The examination of the sections was conducted using light microscopy. As negative controls, normal rabbit IgG or mouse IgG was used to replace the primary antibodies.

For transmission electron microscopy, kidneys were fixed with 2.5% glutaraldehyde in PBS, postfixed with 1% osmium tetroxide in PBS, dehydrated in a graduated series of ethanol dilutions, and embedded in Epon 812 resin. Then blocks were cut using an ultramicrotome (Ultracut; Leica). Ultrathin sections placed on 200-mesh copper were stained with 1% uranyl acetate in 50% ethanol and Reynolds lead citrate. Finally, the prepared sections were examined using a transmission electron microscope (JEM-1230; JEOL, Tokyo, Japan). The assessment of the EM images included the presence and location of glomerular electron-dense deposits(mainly), qualitative assessment of mesangial hypercellularity, endocapillary hypercellularity, podocyte foot process effacement, thickening of the glomerular basement membrane, widen of subendothelial loose layer.

### Detection of immunoglobulins and immune complex levels by enzyme-linked immunosorbent assay

Serum levels of IgA levels were measured through sandwich ELISA method as described previously (7). In brief, plates were coated overnight with 2.5 μg/ml goat F(ab’)_2_ anti-mouse Ig (SouthernBiotech, 1012-01, 1:400) in sodium carbonate buffer (pH 9.6). After washing, the plates were blocked with 1% BSA for 2 hours. Then diluted serum samples and mouse IgA (SouthernBiotech, 0106-01, the first standard concentration is 100ng/ml), used as the standard, were added and incubated for 1 hour. Following another round of washing, the plates were incubated with horseradish peroxidase (HRP)-conjugated goat anti-mouse IgA (SouthernBiotech, 1040-05, 1:5000, 2ug/ml) antibodies for 1 hour. To determine serum levels of IgA-IgG complexes, a cross-capture ELISA was performed. The 96-well plates were coated with 2.5 μg/ml of goat anti-mouse IgA (SouthernBiotech, 1040-01, 1:400) overnight. After washing and blocking, the diluted serum samples were added and allowed to bind for 1 hour. Then the HRP-conjugated goat anti-mouse IgG antibody (SouthernBiotech, 1030-05, 1:5000, 0.2ug/ml) was applied. All reactions were carried out at room temperature. Finally, the plates were developed using 3,3’,5,5’-tetramethylbenzidine (TMB) and the reactions were stopped with 1 M sulfuric acid. The results were analyzed using an ELISA reader (Bio-Rad 550) at 450 and 570 nm wavelengths.

### Biochemical analyses and measurement of blood pressure

Plates were coated with 2.5 μg/ml of goat anti-mouse albumin (BETHYL, A90-134A, 1:400) antibody to detect urine albumin levels. After sample incubating and washing, the HRP-conjugated goat anti-mouse albumin (BETHYL, A90-134P, 1:10000, 0.1ug/ml) was added at 1:10000 dilution for 1 hour. The reaction was developed by adding TMB and stopped with 1 M sulfuric acid. Urinary creatinine levels were determined using a creatinine assay kit (BioAssay Systems, DICT-500). Proteinuria was defined as the urine albumin/creatinine ratio (ACR, mg/g). Hematuria was detected by urine test strips (URIT 1V), which is then scored semi-quantitatively (**Supplementary Figure S1**). Serum creatinine levels were measured using a serum creatinine assay kit (Nanjing Jiancheng Bioengineering Institute, C011-2-1). Blood urea nitrogen levels were assessed using a chemistry kit (BioAssay Systems, DIUR-100). All procedures are the same as the kit instructions. Blood pressure was measured by a noninvasive tail-cuff method (Softron Biotechnology, BP98AWU). One mouse’s blood pressure was measured three times, and the average value was finally calculated.

### Western blotting

Equal amounts of diluted serum were solubilized in SDS sample buffer under nonreducing conditions and subjected to electrophoresis in 4-12% gradient gel to separated monomeric IgA (mIgA) and polymeric IgA (pIgA). The proteins were then transferred to polyvinylidene fluoride membranes (PVDF, Millipore) and subjected to Western blot analysis using HRP-conjugated goat anti–mouse IgA (SouthernBiotech, 1040-05, 1:5000, 0.2ug/ml) antibody. The same method was used for serum C3 detection with HRP-conjugated goat antibodies specific for mouse C3 (MP Biomedicals, 0855557, 1:10000) under reducing conditions. The detection of serum Ba were performed under reducing conditions. And complement factor H (CFH) were detected under nonreducing conditions. Goat anti-mouse FB (Sigma) or CFH (Merck, 341276, 1:5000) antibodies were first incubated on the PVDF membranes followed by HRP-conjugated donkey anti-goat IgG antibody (Abcam, ab97110, 1:10000, 0.1ug/ml). Membranes were developed by enhanced chemical luminescence treatment (GE Healthcare). The grayscale value of Western blotting graphics was quantified by Image J software. Levels of target fragment were reported as fold change relative to control mice.

### Statistics

Normality test is used to determine the distribution of sample data. Continuous variables were presented as the mean ± SD (normally distributed variables) or the median with 25th and 75th centiles (non-normally distributed variables) for data distribution. In this study, we conducted comparisons among three groups - H^W/R^-Buffer, H^W/R^-LCWE^Low^, and H^W/R^-LCWE^High^- under the same genetic background. Furthermore, within the context of the same LCWE dose, we examined two groups: H^W/R^-LCWE^High^ and H^W/W^-LCWE^High^. For comparisons involving two groups of continuous variables, T-tests were employed for normally distributed data, and Mann-Whitney U tests were utilized for non-normally distributed data. When dealing with ordinal categorical variables between two groups, Mann-Whitney U tests were applied. For comparisons related to positive rates in two groups, the chi-square test (specifically Fisher’s exact test) was employed. In cases where three groups were compared for continuous variables, One-way ANOVA was used for normally distributed data, while Kruskal–Wallis test were employed for non-normally distributed data. Ordinal categorical variables, such as IgA and complement C3 deposition intensity, were compared using Kruskal–Wallis test, and positive rates were compared using the chi-square test (Fisher’s exact test). Bonferroni correction was used when three groups are compared.

## Results

### Levels of serum IgA and IgA–IgG immune complexes increase following LCWE injection

**Figure 1:**
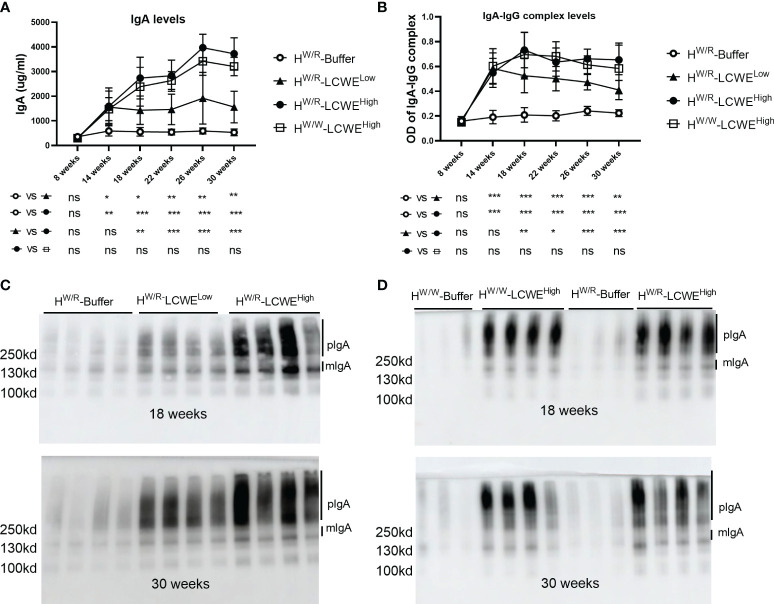
Serum levels of IgA and IgA–IgG complexes. **(A, B)** Quantification of IgA **(A)** and IgA–IgG complexes **(B)** at different time points by ELISA in FH^W/R^-Buffer, FH^W/R^-LCWE^Low^, FH^W/R^-LCWE^High^, FH^W/W^-LCWE^High^. Data are from all mice raised to the 30 weeks old. N=8 mice in each group. **(C, D)** Detection of IgA by western blot in serum. Randomly selected samples of each group. N = 3-4 mice per group. *P < 0.05, **P < 0.01, ***P < 0.001. ns, not significant.

Compared with the PBS group, the body weight of mice induced by LCWE decreased in the first week, but then gradually increased (**Supplementary Figure S2**). IgA levels and IgA-IgG complex levels in mouse sera were measured for up to 30 weeks. As shown in **Figures 1A**, **B**, both IgA and IgA-IgG levels were elevated in the LCWE groups at 14 weeks of age, two weeks after the completion of LCWE injections at 12 weeks. Over the next 16 weeks, IgA and IgA-IgG levels stayed significantly higher in all dose groups of LCWE as compared to those in the buffer group (*P* < 0.05). Among FH^W/R^ mice, there was a clear correlation between the LCWE doses and the levels of circulating IgA or IgA-IgG after 14 weeks of age (*P* < 0.05). Meanwhile, there was no difference in IgA levels and IgA-IgG levels between FH^W/R^ and FH^W/W^ in the high LCWE dose group.

In addition, we measured the level of high molecular weight polyIgA (pIgA) (**Figures 1C, D** and **Supplementary Figure S3**). Both pIgA and mIgA levels in the FH^W/R^-LCWE^High^ group were higher than those in the FH^W/R^-LCWE^Low^ group (*P* < 0.05). This is consistent with polymeric to monomeric IgA ratios that appear also higher in the FH^W/R^-LCWE^High^ group at 18 weeks, albeit this has not reached statistical significance (**Supplementary Figure S3A**, **2C**). When FH^W/R^- LCWE^High^ and FH^W/W^- LCWE^High^ were compared, there was no significant difference in pIgA levels at 18 weeks and 30 weeks (**Figure 1D**). We observed a slightly higher level of mIgA in the FH^W/R^-LCWE^High^ group, with low poly-to-mono-IgA ratios at 18 weeks (**Supplementary Figure S3B**).

### The FH^W/R^- LCWE^High^ group exhibits strong mesangial IgA and C3 deposition

**Figure 2:**
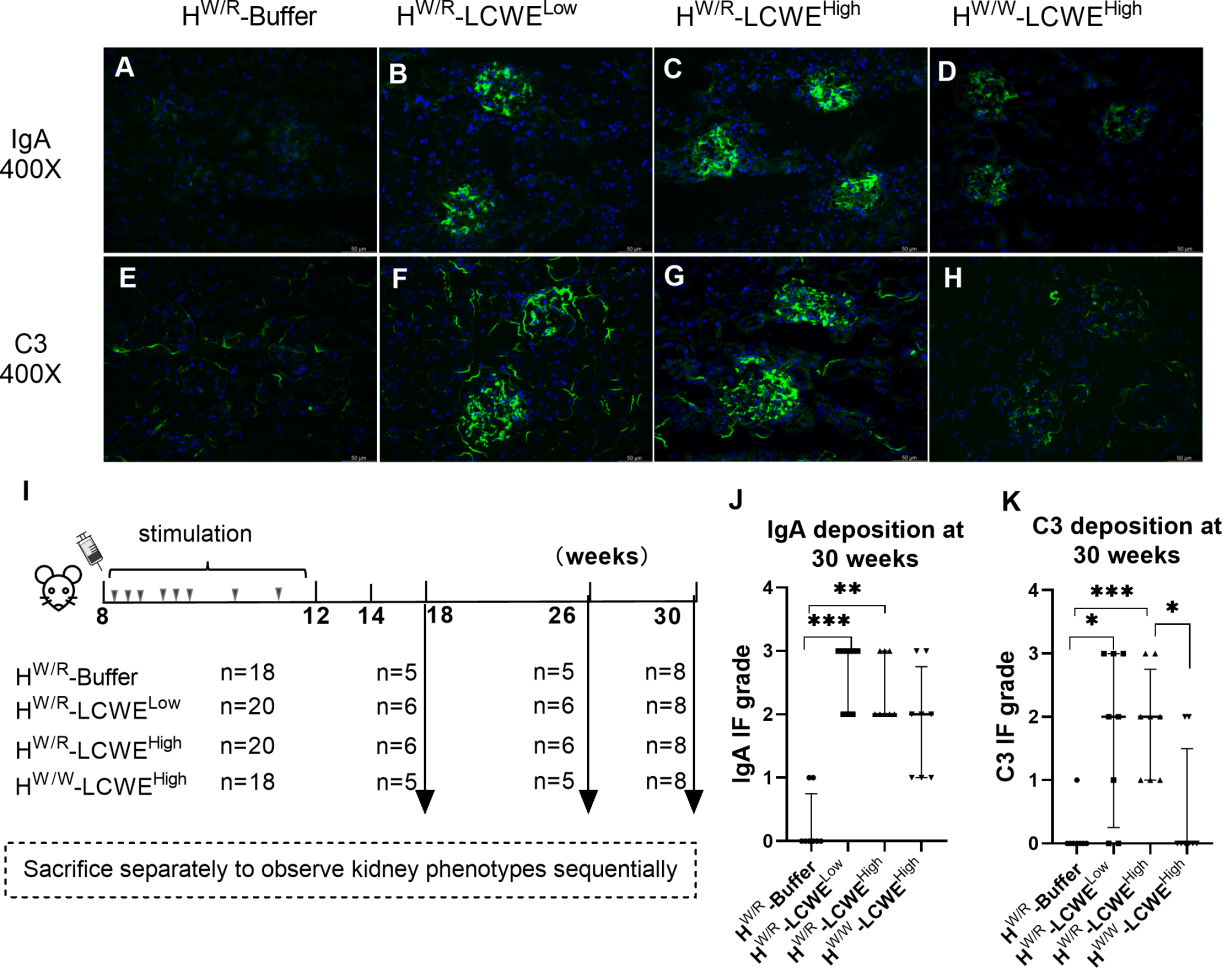
Immunofluorescence staining of IgA and C3 in kidneys. **(A–D)** Representative images of IgA in every groups. **(E–H)** Representative images of C3. **(I)** After LCWE induction, mice were sacrificed at different times. **(J–K)** Semi-quantitative scoring of IgA and C3 fluorescence intensity. Mann-Whitney U tests and Kruskal–Wallis test (Bonferroni correction) were used when comparing between two groups and between three groups respectively. Data are from all mice raised to the 30 weeks old. N = 8 mice per group. Scale bars: 50 μm. *P < 0.05, **P < 0.01, ***P < 0.001.

We detected IgA and C3 in the kidneys by immunofluorescence staining (**Figures 2A–H**). In order to observe the renal phenotype of mice sequentially, we sacrificed mice at 18 weeks, 26 weeks, and 30 weeks respectively (**Figure 2I**). All mice primed with LCWE had IgA signals in the glomerulus. By contrast, only 22.2% of the FH^W/R^-Buffer group (4/18) showed IgA positivity, all with relatively low signal intensity. And at 30 weeks, the positivity rate of the FH^W/R^-Buffer group is 25% (**Figure 2J**). Regarding C3, the FH^W/R^-LCWE^High^ group had an overall positive rate of 90% (18/20), which reached 100% by 30 weeks of age (8/8). In the FH^W/R^-LCWE^Low^ group, C3 positivity was 70% (14/20), climbing to 75% (6/8) by 30 weeks. Importantly, the FH^W/W^-LCWE^High^ group exhibited a relatively lower C3 positive rate (2/8) despite all having IgA deposition. These findings indicate that factor H mutation in FH^W/R^ promotes C3 co-deposition with IgA in the glomeruli (**Supplementary Figures S4A, B, C**), consistent with the role for factor H that as a potent inhibitor of AP.

We also observed interesting differences in the deposition patterns of IgA and C3. In the FH^W/R^-LCWE^Low^ and FH^W/W^-LCWE^High^ groups, the majority of mice displayed diffuse deposition of IgA and C3 in the mesangial area (**Figures 2B, C**). However, FH^W/R^-LCWE^High^ mice displayed varying patterns of deposition, ranging from diffuse mesangial deposition, with or without additional deposits in the capillary loop (**Supplementary Figure S4D**). Furthermore, mice with more severe glomerular lesions tended to have granular deposits of IgA (**Supplementary Figure S4G**).

### Factor H heterozygous mice have more severe lesions

**Figure 3:**
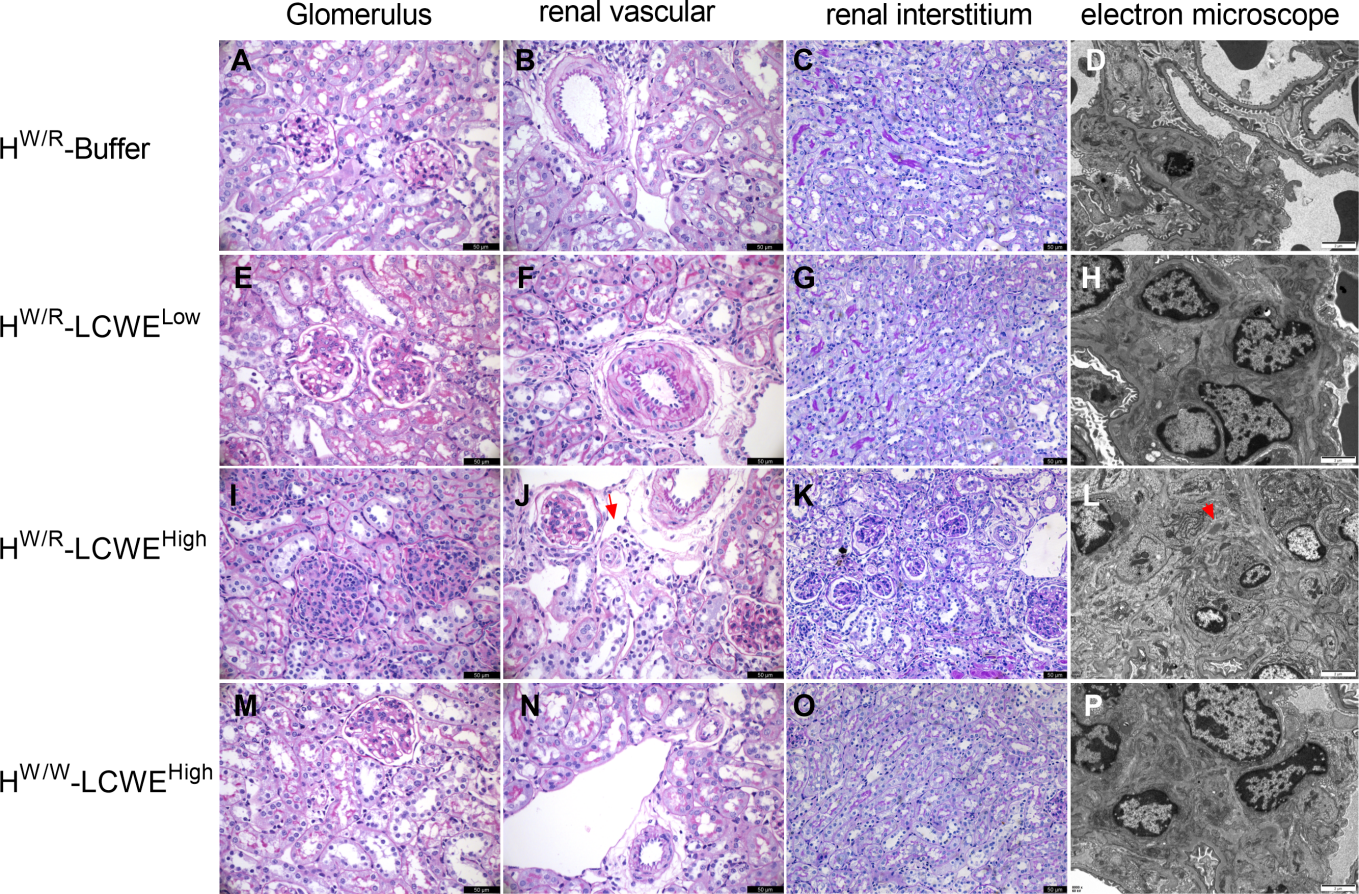
Representative micrographs of PAS staining and electron micrographs from each group are shown. **(A–D)** Normal glomerulus, renal vascular and renal interstitium from FH^W/R^-Buffer group mice. **(E–H)** Mice in FH^W/R^-LCWE^Low^ group showed mesangial hypercellularity, and the arterioles from a mouse showed thickening of arteriolar walls and stenosis of lumens. **(I–L)** Mice from FH^W/R^-LCWE^High^ group had diverse lesions, including mesangial hypercellularity and endothelial hypercellularity **(I)**, arteriolar onion-like lesions (**J**, the red arrow), tubular atrophy/interstitial fibrosis **(K)**. Mice exhibited mesangial cell proliferation and marked mesangial expansion. Electron-dense deposits were apparent in the mesangial area (**L**, the red arrow). **(M-P)** FH^W/W^-LCWE^High^ group mice showed milder pathological lesions compared with FH^W/R^-LCWE^High^ group mice. Lesions are mainly mild mesangial hypercellularity **(M)**. In the PAS staining image, the magnification of the renal interstitium is 200X and the rest is 400X. Scale bars in PAS figures: 50 μm. Scale bars in electron micrographs: 2μm.

As expected, FH^W/R^-Buffer mice all had normal kidney histology (**Figures 3A–C**), whereas the FH^W/R^-LCWE^Low^ group developed glomerular and vascular lesions (**Figures 3E–G**). The glomerular lesions predominantly manifested as mesangial hypercellularity (M1) based on Oxford classification of IgAN. And mesangial cell proliferation and electron-dense deposition can be seen under electron microscopy (**Figure 3H**). In the FH^W/R^-LCWE^High^ group, in addition to M1, the majority of animals also presented with endocapillary hypercellularity (E1, 17/20, 85%) (**Figure 3I**), with some showing crescent formation (C1, 3/20, 15%) (**Supplementary Figures S4E, H**), and tubular atrophy/interstitial fibrosis (T1, 4/20, 20%) (**Figure 3K**). Consistent with clinical IgAN that frequently has arteriolar lesions, 35% (7/20) FH^W/R^-LCWE^High^ mice developed arteriolar lesions, with 2 mice showing onion-like lesions (10%) (Detailed information is in **Table 1**) (**Figure 3J**). In addition, we found no mice had microthrombi in glomerular capillary lumen and renal arteriolar thrombosis (0/20). Overall, mesangial hypercellularity is the dominant type of lesion of the FH^W/R^-LCWE^Low^ group, whereas FH^W/R^-LCWE^High^ animals exhibited a greater variety of lesion types, indicating severity of kidney damage. A comparison between FH^W/R^-LCWE^Low^ and FH^W/R^-LCWE^High^ groups revealed pathological severity in correlation with LCWE dose.

**Table 1:** Pathological lesions of all mice from four groups.

	M1	E1	S1	T1	C1	A1
H^W/R^-Buffer (n=18)	3 (16.7%)	0	0	0	0	0
H^W/R^-LCWE^Low^ (n=20)	19 (95%)	3 (15%)	0	0	0	2 (10%)
H^W/R^-LCWE^High^ (n=20)	20 (100%)	17 (85%)	3 (15%)	4 (20%)	3 (15%)	7 (35%)
H^W/W^-LCWE^High^ (n=18)	14 (77.8%)	2 (11.1%)	0	0	0	1 (5.6%)

Pathological lesions were evaluated using PAS staining with reference to the Oxford classification of IgA nephropathy. M, Mesangial hypercellularity; E, Endocapillary hypercellularity; S, Segmental glomerulosclerosis; T, Tubular atrophy/interstitial fibrosis; C, Cellular/fibrocellular crescents; A, Arteriolar lesions.

Interestingly, FH^W/W^-LCWE^High^ had only mild mesangial hypercellularity (M1,14/18), with 2 out of 18 mice having endocapillary hypercellularity (E1) and 1 mouse had arteriolar lesions (A1) (**Figures 3M–P**). In contrast, FH^W/R^-LCWE^High^ animals exhibited a greater diversity and severity of glomerular lesions, suggesting complement activation contributes to glomerular damage.

Transmission electron microscopy (TEM) of FH^W/R^-Buffer kidneys showed normal basement membranes and podocyte foot processes. No evidence of mesangial cell proliferation or other glomerular injuries was observed (**Figure 3D**). In contrast, mice that had been primed with LCWE, particularly those in the FH^W/R^-LCWE^High^ group, exhibited mesangial cell proliferation and marked mesangial expansion (**Figure 3L**). Electron-dense deposits were apparent in the mesangial area across all three dose groups, and leukocyte infiltration (neutrophils) was observed in the capillary loops of some LCWE-primed mice (**Supplementary Figure S4F**). In addition, foot process effacement was observed in FH^W/R^-LCWE^High^ mice, indicating severity of the lesions (**Supplementary Figure S4I**).

### FH^W/R^-LCWE^High^ mice developed proteinuria and hematuria

**Figure 4:**
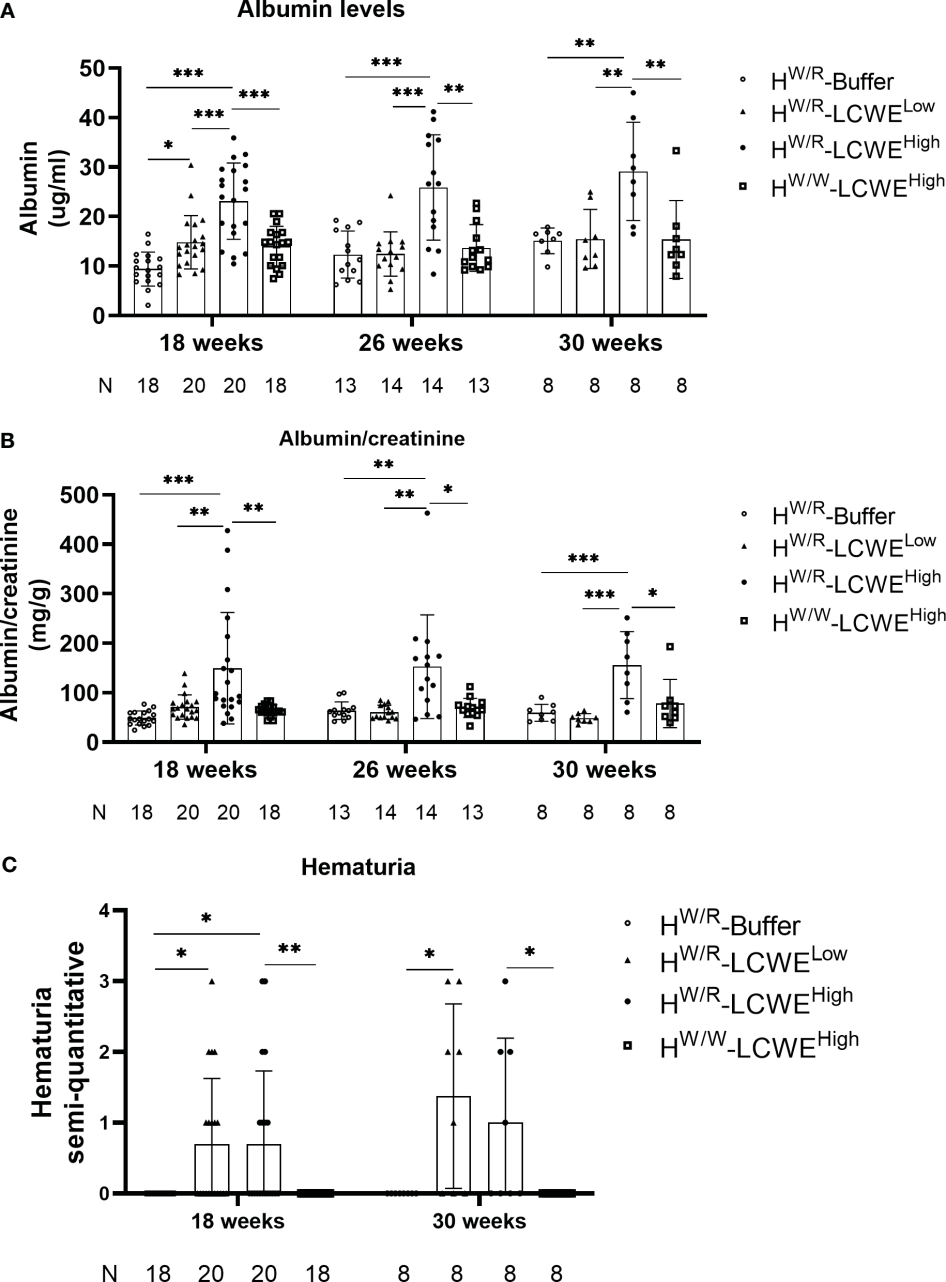
Kidney function analysis of all the mice. **(A–C)** The albumin levels and hematuria degree from all mice at different time point. Each point represents 1 mouse. The A depicted the results of urinary albumin and the B showed the urinary albumin normalized to creatinine. T-tests and one-way ANOVA (Bonferroni correction) were used when comparing between two groups and between three groups respectively. C shows the results of hematuria. Mann-Whitney U tests and Kruskal–Wallis test (Bonferroni correction) were used when comparing between two groups and between three groups respectively.

We detected urinary albumin levels in mice at 18, 26, and 30 weeks of age (**Figures 4A, B**). Consistent with more severe pathological damage, the FH^W/R^-LCWE^High^ group also had elevated levels of urinary albumin. At 18 weeks old of mice, two weeks after the completion of LCWE injections, urine albumin levels were higher in the FH^W/R^-LCWE^High^ group than in the FH^W/R^-Buffer, in the FH^W/R^-LCWE^Low^, and in the FH^W/W^-LCWE^High^ group (23.11 ± 7.72 *vs* 9.39 ± 3.44 *vs* 14.8 ± 5.41 *vs* 14.01 ± 4 mg/g, *P*<0.05, **Figure 4A**). After correcting for urine creatinine, FH^W/R^-LCWE^High^ group is still higher than other groups (*P*<0.05, **Figure 4B**).

We also measured hematuria using urine test strips. At 18 weeks and 30 weeks of age, approximately half of the mice in both FH^W/R^-LCWE^High^ and FH^W/R^-LCWE^Low^ groups had hematuria (**Figure 4C**). Whereas none of the FH^W/W^-LCWE^High^ mice exhibited hematuria, indicating the critical contribution of complement activation in the progression of the disease.

We tested serum creatinine and BUN levels in serum samples of mice raised to 30 weeks of age. As shown in **Supplementary Figure S5**, mice in the FH^W/R^-LCWE^High^ group exhibited higher serum BUN levels compared to those in the FH^W/R^-Buffer and FH^W/R^-LCWE^Low^ group. Notably, no statistically significant difference was found when compared to mice in the FH^W/W^-LCWE^High^ group (*P*=0.054). However, no statistically significant difference was observed in serum creatinine levels among the four groups.

Blood pressure was assessed at 30 weeks of age, and as illustrated in **Supplementary Figure S6**, no significant differences were observed in systolic blood pressure (108 ± 10 vs. 103 ± 9 vs. 94 ± 15 vs. 100 ± 15 mmHg), diastolic blood pressure (44 ± 4 vs. 46 ± 7 vs. 41 ± 13 vs. 54 ± 19 mmHg), and mean arterial pressure (65 ± 6 vs. 63 ± 8 vs. 58 ± 13 vs. 52 ± 8 mmHg) among the four groups of mice.

### Glomerular complement activation in FH^W/R^-LCWE^High^ mouse

**Figure 5:**
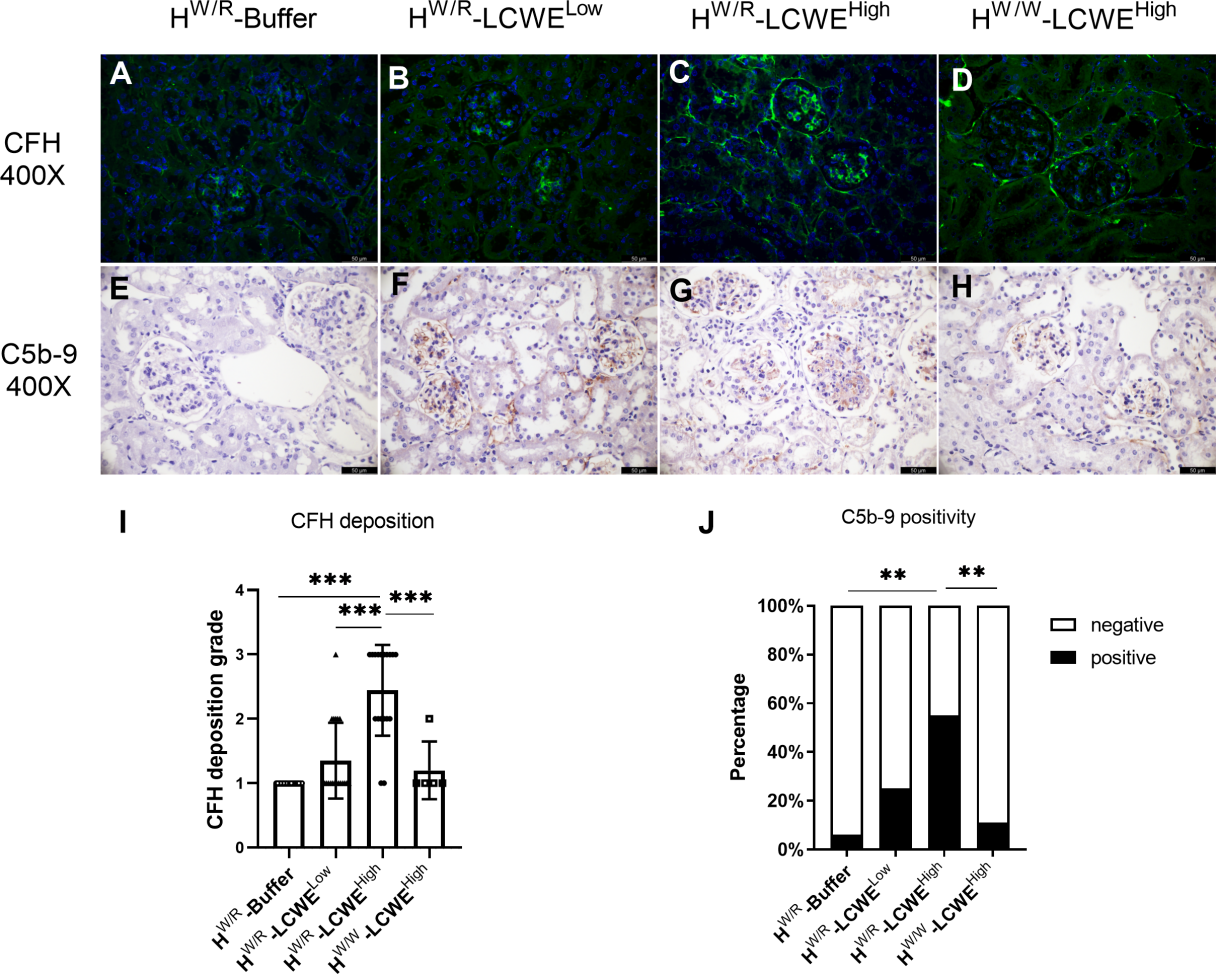
The deposition of complement C5b-9 and H in the kidney. **(A–D)** Representative micrographs of CFH staining from each group are shown. **(E–H)** Representative micrographs of C5b-9 staining from each group. Mice in FH^W/R^-LCWE^High^ group showed more FH deposition and more C5b-9 activation **(I, J)** For H, Mann-Whitney U tests and Kruskal–Wallis test (Bonferroni correction) were used when comparing between two groups and between three groups respectively. For C5b-9, Fisher’s exact test was used. Scale bars: 50 μm. **P < 0.01, ***P < 0.001.

Co-deposition of C3 with IgA was observed in the glomerulus (**Supplementary Figure S4C**). FH^W/R^-LCWE^High^ mice had higher C3 positivity than their FH^W/W^-LCWE^High^ counterparts (100% vs. 25%, **Figure 2K**) at 30 weeks, consistent with the expectation of the factor H heterozygous mice being more susceptible to complement activation. While factor H signals were weak in FH^W/R^-Buffer animals, the addition of LCWE priming significantly enhanced factor H deposition in the kidney. This appeared more prominently in the FH^W/R^-LCWE^High^ group than in the FH^W/W^-LCWE^High^ and the FH^W/R^-LCWE^Low^ groups (**Figures 5A–D**, **I**). These results indicate the dynamics of complement activation in response to IgA deposition being driven by inflammation, whereas factor H counteracts the overactivation of C3.

Furthermore, C5b-9 participates in the membrane attack complex (MAC) in the final step of complement activation. We performed immunohistochemistry (IHC) to detect C5b-9 in the kidney (**Figures 5E–H**). In the FH^W/R^-Buffer group, only 1 out of 18 mice had weak signals of C5b-9. Similarly, in the FH^W/W^-LCWE^High^ group, only 2 out of 18 mice showed weak positivity. Meanwhile, C5b-9 positive rate increased to 25% (5/20) in the FH^W/R^-LCWE^Low^ group, illustrating the importance of FH in suppressing complement activity. Notably, C5b-9 positive rate in the FH^W/R^-LCWE^High^ group reached 55% (11/20) (**Figure 5J**). FH^W/R^-LCWE^High^ mice that exhibited S1, T1 or C1 lesions generally had a higher C5b-9 deposition rate (5/5) as compared to the mice with just M1 or M1/E1 lesions (6/15). This finding indicates that more severe lesions were associated with increased complement activation.

To better understand the complement pathways involved in the renal damage, we performed immunofluorescence staining of C1q (representing the classical pathway), Bb (representing the alternative pathway) and MBL (representing the lectin pathway) using frozen sections of kidney tissue (**Supplementary Figure S7**). The C1q positivity rate is low, only 2 and 1 mice were found to have weak positive deposition of C1q in H^W/R^-LCWE^High^ group (2/20) and H^W/W^-LCWE^High^ group (1/18) respectively. **Supplementary Figure S7A** shows that one mouse in H^W/R^-LCWE^High^ group is weakly positive for C1q. Under the induction of LCWE, 2 mice in H^W/R^-LCWE^Low^ group (2/20), 4 mice in H^W/R^-LCWE^High^ group (4/20) and 2 mice in H^W/W^-LCWE^High^ group (2/18) showed MBL positive (**Supplementary Figure S7B**). The overall MBL positive rate was not high and there was no statistical difference between the groups (**Supplementary Figure S7H**). In contrast, the activation of the alternative pathway in H^W/R^-LCWE^High^ group is stronger. 1 mouse in H^W/R^-Buffer group, 3 mice in H^W/R^-LCWE^Low^ group (3/20), 15 mice in H^W/R^-LCWE^High^ group (15/20) and 3 mice in H^W/W^-LCWE^High^ group (3/18) showed Bb positive (**Supplementary Figures S7C, D**). The Bb positive rate of H^W/R^-LCWE^High^ group was significantly higher than that of the other three groups (**Supplementary Figure S7I**).

### FH^W/R^-LCWE^High^ mice exhibit complement activation in circulation

**Figure 6:**
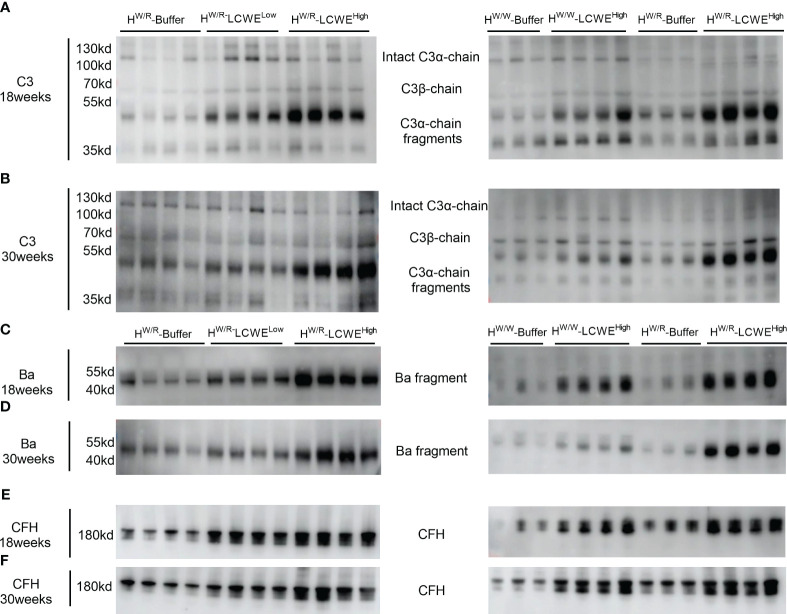
Complement activation in circulation. **(A, B)**: western blot of C3 at 18 weeks and 30 weeks respectively. **(C, D)**: western blot of Ba at 18 weeks and 30 weeks respectively. **(E, F)**: western blot of FH at 18 weeks and 30 weeks respectively.

Next, we performed Western blotting to evaluate the status of complement activation in circulation by measuring the ratio between intact C3α-chain and the resulting C3α-chain fragments following complement activation. At 18 weeks (**Figure 6A**) and 30 weeks (**Figure 6B**), the FH^W/R^-LCWE^High^ group showed higher levels of cleaved C3α fragments than the FH^W/R^-LCWE^Low^ group (*P* < 0.05, **Supplementary Figures S8A, B**). The difference in ratios had reached statistical differences at 30 weeks (**Supplementary Figure S8F**). And the FH^W/R^-LCWE^High^ group showed higher C3α-chain fragments/intact C3α-chain ratio than FH^W/W^-LCWE^High^ group at 18 weeks and 30 weeks (*P* < 0.05, **Supplementary Figures S8G, H**). Overall, the FH^W/R^-LCWE^High^ group had the highest active fragments to intact C3α ratio, indicating complement activation.

In addition to C3, we performed Western blotting of factor H (FH) and factor B (FB) of the alternative pathway. FH^W/R^-LCWE^High^ had higher circulating factor H levels than FH^W/R^-LCWE^Low^ (**Figure 6** and **Supplementary Figures S8I, J**). However, there was no statistical difference in factor H levels between the FH^W/R^-LCWE^High^ group and the FH^W/W^-LCWE^High^ group at 18 weeks and 30 weeks.

With respect to factor B, we measured factor B cleavage fragment Ba. Our findings were consistent with those for the C3α-chain cleavage fragments. The levels of Ba were elevated in the FH^W/R^-LCWE^High^ group as compared to the FH^W/R^-Buffer and FH^W/R^-LCWE^Low^ groups. Moreover, FH^W/R^-LCWE^High^ animals exhibited higher Ba levels than their FH^W/W^-LCWE^High^ counterparts, consistent with the notion that FH mutant mice are more predisposed to complement activation.

## Discussion

We have previously developed a continuous and stable IgA deposition model induced by LCWE, which partially replicates the upstream mechanisms (hits 1, 2, and 3) in IgA nephropathy. This model exhibits increased intestinal permeability, elevated levels of IgA and IgA-IgG, as well as mesangial IgA deposition, accompanied by partial C3 deposition. However, the pathological manifestations of this model are relatively mild and limited, as these mice do not present proteinuria or hematuria. In the current study, we investigated factor H heterozygous mutant mice, which have a genetic basis that predisposes them to complement activation. With the addition of LCWE stimulation, these mice developed severe and diverse pathological damages in the glomerulus, closely resembling the characteristics of IgA nephropathy. There was no overmortality and mice at 30 weeks were morphologically not substantially different from control mice.

Complement activation through the alternative pathway plays a crucial role in the pathogenesis of IgA nephropathy (1, 11). Numerous studies conducted over the years have discovered elevated circulating levels and mesangial deposition of complement proteins of the alternative pathway (12–23). Additionally, and most importantly, large genome-wide association studies (GWAS) of IgA nephropathy have consistently detected the susceptible locus 1q32, which contains the CFH and CFHRs genes (8) (24, 25). In CFH and CFHRs, studies had found that there are regulatory genetic variations affecting the expression levels of circulating complement proteins (26, 27), and there are also genetic variations in the coding region affecting protein structure (26, 28). All of these studies jointly revealed the important roles of CFH and CFHRs in regulating complement activation of the alternative pathway through multiple mechanisms and influence the severity of renal tissue damage. In this study, we developed mouse model based on factor H heterozygous mutant mice. By comparing and FH^W/W^-LCWE^High^ mice under the same dose induction, we discovered that the FH^W/R^-LCWE^High^ group developed more severe and diverse lesions and had higher levels of proteinuria, more pronounced complement activation in the kidney. This observation indicates alternative pathway activation contributes to the aggravation of pathological damage. Numerous studies emphasize the close association between factor H-related protein and IgA nephropathy. A mouse model based on humanized factor H-related protein may be better able to understand the role of alternative complement pathway in human IgAN.

Our model also corroborates our previous findings of both IgA levels and IgA-IgG complex levels being elevated after mucosal inflammation (7). And in this study, we conducted a comparative analysis between FH^W/R^-LCWE^High^ group mice and FH^W/R^-LCWE^Low^ group mice, which revealed that high-dose LCWE had a more pronounced effect compared to low-dose LCWE. Specifically, FH^W/R^-LCWE^High^ group mice exhibited higher IgA levels and IgA-IgG levels, as well as more severe pathological lesions in the kidneys. This was attributed to higher levels of IgA and IgA-IgG complexes on the one hand, and higher levels of mutant factor H on the other hand.

Intrarenal arterial lesions are common in patients with IgAN. Prior studies, including our own cohort study, consistently demonstrated that arteriolar microangiopathic lesion(MA) was an independent risk factor for kidney failure (29) and suggested a potential involvement of complement activation in the development of arteriole lesions, especially those with MA (15, 16, 29). In previously published studies, mice with homozygous mutations in factor H exhibited aHUS, a type of thrombotic microangiopathy (TMA) while mouse with heterozygous mutant didn’t show arteriolar lesions (10). In this study, we observed that 35% of the mice in the FH^W/R^-LCWE^High^ group developed renal arteriolar lesions, with some exhibiting concomitant MA. In contrast, FH^W/W^-LCWE^High^ mice did not have MA, suggesting the alternative complement activation after IgA kidney deposition contribute to the endothelial lesions.

Our study demonstrates that complement activation after IgA kidney deposition contribute to the renal phenotype in mice. Mutation in the complement system might render indolent IgA deposits pathologic. Our model is closer to the four-hit theory to a certain extent, it may help to advance our knowledge of IgAN and may be a useful tool for preclinical evaluation of treatment response to complement-inhibitors. Also it should be cautious that our present model cannot fully reflect and represent human IgAN for not all patients have complement gene mutations.

In summary, our study established a new animal model of IgAN, highlighting the significant role of complement activation in the pathogenesis of the disease. This model will serve as a valuable tool for advancing research on IgAN towards the understanding of complement-mediated renal damage. The new model also provides a useful tool for the development of therapies for IgAN.

## Data availability statement

The raw data supporting the conclusions of this article will be made available by the authors, without undue reservation.

## Ethics statement

The animal study was approved by Laboratory Animal Care and Use Committee of Peking University First Hospital. The study was conducted in accordance with the local legislation and institutional requirements.

## Author contributions

JyL: Data curation, Formal analysis, Investigation, Methodology, Resources, Software, Validation, Visualization, Writing – original draft. YD: Data curation, Investigation, Methodology, Writing – original draft. FC: Data curation, Resources, Writing – original draft. HY: Funding acquisition, Supervision, Writing – review & editing. PC: Funding acquisition, Supervision, Validation, Writing – review & editing. HL: Investigation, Writing – review & editing. SS: Supervision, Writing – review & editing. XZ: Supervision, Writing – review & editing. LZ: Supervision, Writing – review & editing. YZ: Supervision, Writing – review & editing. LL: Supervision, Writing – review & editing. XX: Supervision, Writing – review & editing. FY: Resources, Supervision, Writing – review & editing. JJ: Project administration, Supervision, Writing – review & editing. JcL: Conceptualization, Funding acquisition, Project administration, Supervision, Writing – review & editing. HZ: Conceptualization, Funding acquisition, Project administration, Supervision, Writing – review & editing.
