# Supplementary material for: Heterozygous mutations in factor H aggravate pathological damage in a stable IgA deposition model induced by Lactobacillus casei cell wall extract
Source: Front Immunol. 2024 Mar 15;15:1368322. doi: 10.3389/fimmu.2024.1368322 (PMC10978756; doi:10.3389/fimmu.2024.1368322)
Supplement: Supplementary file 1 [file DataSheet_1.pdf]

Supplementary materials:

**Supplementary Table S1**	Pathological classification
**Supplementary Figure S1**	Semi quantitative score of hematuria.
**Supplementary Figure S2**	Weight changes of mice.
**Supplementary Figure S3**	mIgA, pIgA and pIgA-mIgA ratio levels (densitometry estimation of western blot).
**Supplementary Figure S4**	Immunofluorescent, light, and electron microscopy of kidneys from $FH^{W/R}$-$LCWE^{High}$ group mice.
**Supplementary Figure S5**	Serum creatinine and BUN levels.
**Supplementary Figure S6**	Blood pressure of mice.
**Supplementary Figure S7**	Complement C1q, MBL and Bb deposition on glomeruli.
**Supplementary Figure S8**	The level of C3α-chain cleavage fragments, C3α-chain fragments/C3α ratio, FH and Ba fragments by densitometry estimation of western blot.

Supplementary Table S1. Pathological classification

**key pathologic features**	**Description**
M	Mesangial score <0.5 (M0) or >0.5 (M1)
E	Endocapillary hypercellularity absent (E0) or present (E1)
S	Segmental glomerulosclerosis absent (S0) or present (S1); presence or absence of podocyte hypertrophy/tip lesions in biopsy specimens with S1
T	Tubular atrophy/interstitial fibrosis ≤25% (T0), 26%–50% (T1), or >50% (T2)
C	Cellular/fibrocellular crescents absent (C0), present in at least 1 glomerulus (C1), in >25% of glomeruli (C2)
A	Arteriolar lesions present, including arteriolar wall thickening, onion skin lesions

The pathological lesions (MEST-C) of mice were scored according to the Oxford classification of IgA nephropathy. In addition, we evaluated arteriolar lesions (A) including wall thickening and onion-like lesions.

Supplementary Figure S1.

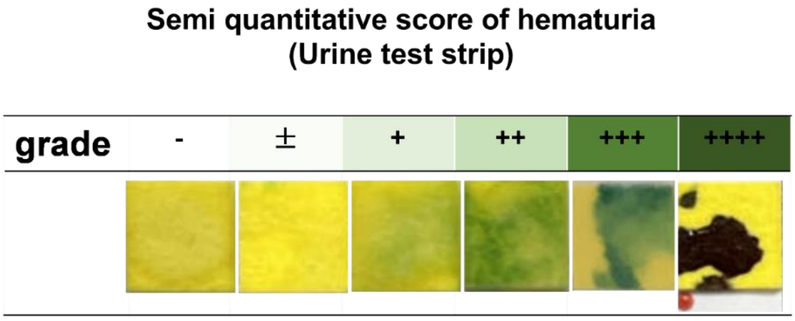

Semi quantitative score of hematuria. The intensity of hematuria was evaluated and scored on a scale of - to ++++: -, no hematuria; ±, trace; +, mild hematuria; ++, moderate hematuria; and +++, strong hematuria; ++++ was gross hematuria.

Supplementary Figure S2.

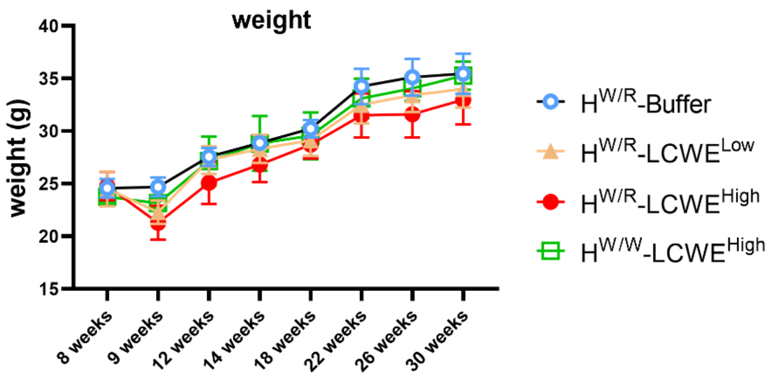

Weight changes of mice in each group (n=8 in each group).

Supplementary Figure S3.

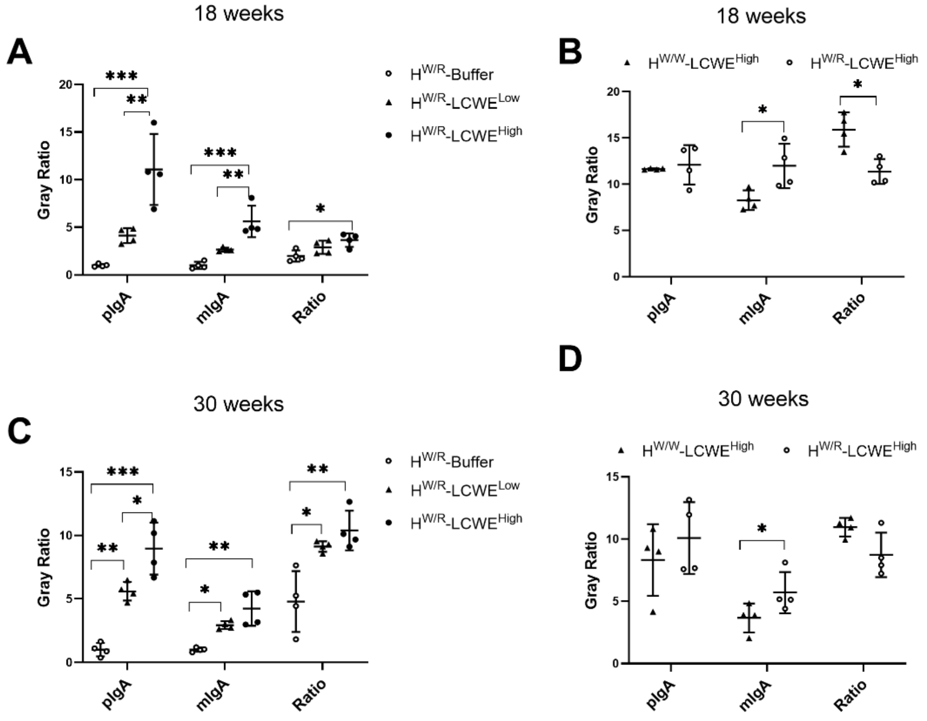

mIgA, pIgA and pIgA-mIgA ratio levels (Densitometry estimation of western blot). A and C: Western blot analysis of serum IgA showing mice of FH$^{W/R}$-LCWE^{High} group have higher pIgA, mIgA and pIgA-mIgA ratio at 18 weeks (A) and 30 weeks (C). One-way ANOVA (Bonferroni correction) were used. B and D: Western blot analysis of serum IgA of F FH$^{W/R}$-LCWE^{High} group and FH$^{W/W}$-LCWE^{High} group at 18 weeks (B) and 30 weeks (D). T-test was used. *P < 0.05, **P < 0.01

Supplementary Figure S4.

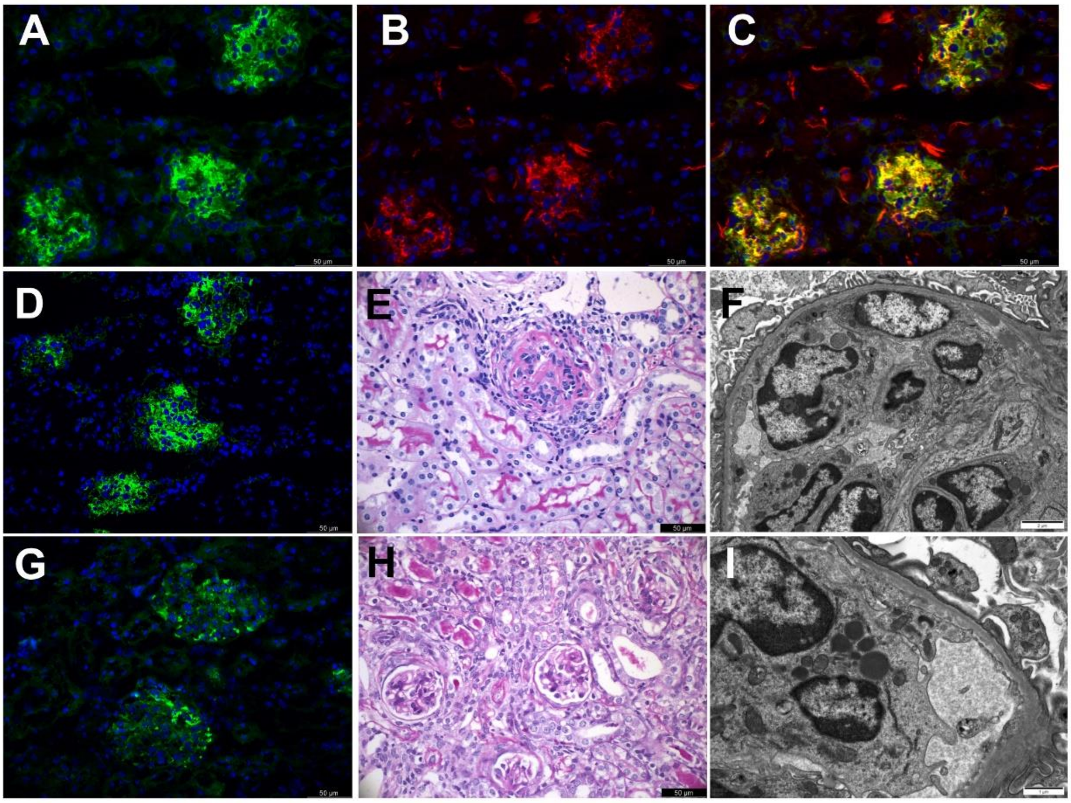

Immunofluorescent, light, and electron microscopy of kidneys from FH$^{W/R}$-LCWE^{High} group mice. The C showed the co-deposition of IgA (A) with C3 (B). The D and G showed the different deposition pattern of IgA. The D exhibited deposition in both the mesangial area and capillary loops and the G showed granular deposition of IgA. Scale bars: 50 μm. The E and H showed the crescent lesions. Scale bars: 50 μm. The F and I exhibited the leukocyte infiltration (neutrophils) and effaced podocyte foot processes. Scale bars: 2 μm and 1 μm, respectively.

Supplementary Figure S5.

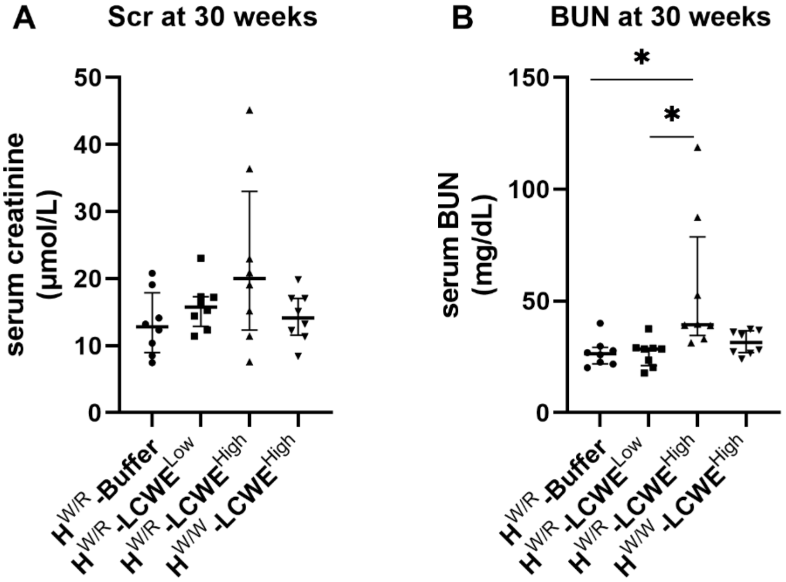

Serum creatinine and BUN levels. Data came from the mice raised to 30 weeks of age. A showed that there is no statistical difference in serum creatinine levels between the four groups. B showed that, compared with the mice in $FH^{W/R}$-Buffer and $FH^{W/R}$-$LCWE^{Low}$ group, mice in $FH^{W/R}$-$LCWE^{High}$ group had higher serum BUN levels. T-tests and one-way ANOVA (Bonferroni correction) were used when comparing between two groups and between three groups respectively. *$P < 0.05$.

Supplementary Figure S6.

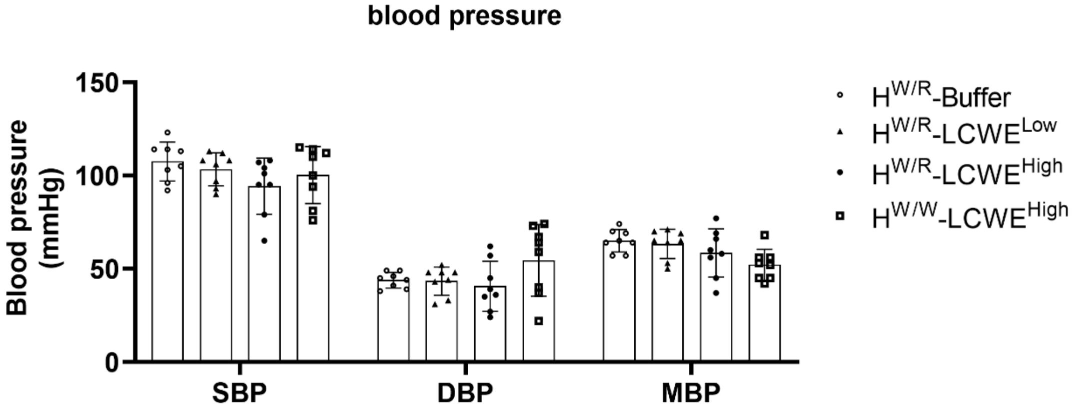

Blood pressure of mice. There was no significant difference in systolic blood pressure, diastolic blood pressure, and mean arterial pressure among the four groups of mice. T-tests and one-way ANOVA (Bonferroni correction) were used when comparing between two groups and between three groups respectively.

Supplementary Figure S7.

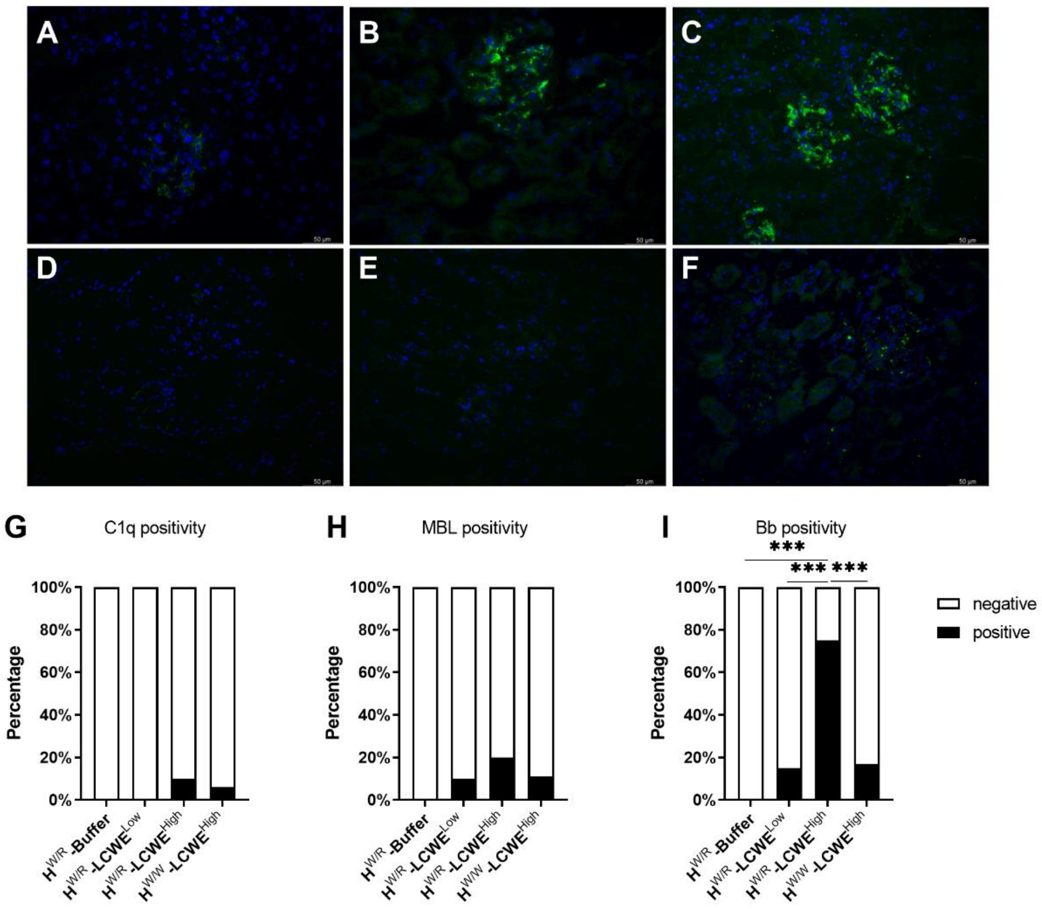

Complement C1q, MBL and Bb deposition on glomeruli. A, B and C are representative images of glomeruli that are weakly positive for C1q, positive for MBL and Bb respectively. C and D show the C1q- and MBL-negative deposition in the glomerulus, respectively. F shows granular deposition of Bb on glomeruli. The C1q and MBL positivity rate is low (G and H). In contrast, the activation of the alternative pathway in $H^{W/R}$-$LCWE^{High}$ group is stronger (I). ****P* < 0.001. Fisher's exact test (Bonferroni correction) was used.

Supplementary Figure S8.

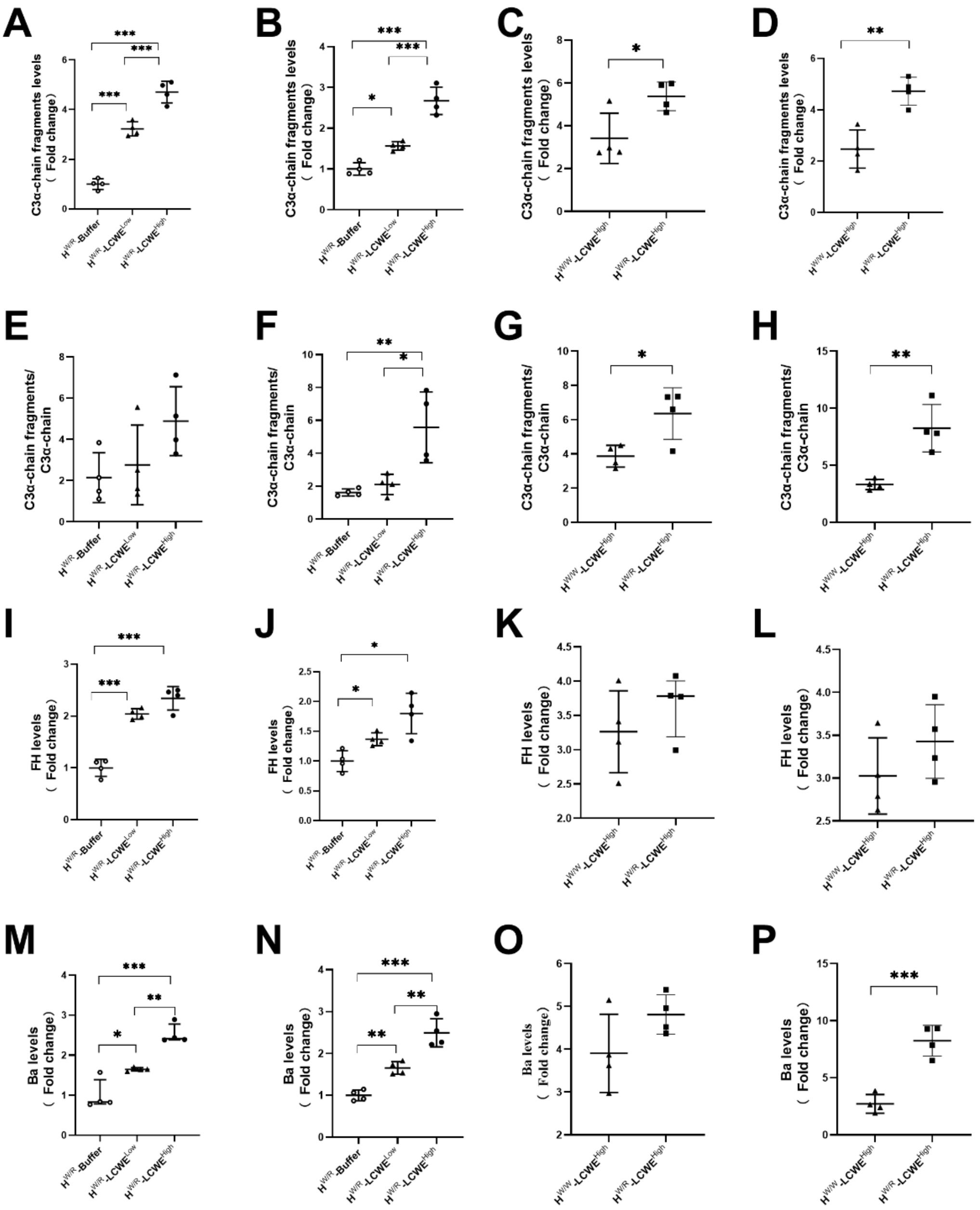

The level of C3α-chain cleavage fragments, C3α-chain fragments/C3α ratio, FH and Ba fragments by densitometry estimation of western blot. n=4, each group in the figure. The first and third columns represent 18 weeks (A, E, I, M, C, G, K, O). The second and fourth columns represent 30 weeks (B, F, J, N, D, H, L, P). T-tests and one-way ANOVA (Bonferroni correction) were used when comparing between two groups and between three groups respectively. **P* < 0.05, ***P* < 0.01, ****P* < 0.001
